# The Role of Platelet Microparticle Associated microRNAs in Cellular Crosstalk

**DOI:** 10.3389/fcvm.2018.00029

**Published:** 2018-04-04

**Authors:** Luoxing Xia, Zhi Zeng, Wai Ho Tang

**Affiliations:** Institute of Pediatrics, Guangzhou Women and Children's Medical Centre, Guangzhou Medical University, Guangzhou, China

**Keywords:** platelet, microparticle, microRNA, RNA, cardiovascular disease

## Abstract

Platelet is an anucleate cell containing abundant messenger RNAs and microRNAs (miRNAs), and their functional roles in hemostasis and inflammation remain elusive. Accumulating evidence has suggested that platelets can actively transfer RNAs to hepatocytes, vascular cells, macrophages, and tumor cells. The incorporated mRNAs are translated into proteins, and miRNAs were found to regulate the gene expression, resulting in the functional change of the recipient cells. This novel intercellular communication opens up a new avenue for the pathophysiological role of platelet in platelet-associated vascular diseases. Therefore, understanding the underlying mechanism and identification of the platelet miRNAs involved in this biological process would provide novel diagnostic and therapeutic targets for cardiovascular diseases.

## Introduction

Platelet plays a pivotal role in maintaining primary hemostasis, and contributes to the pathogenesis of thrombotic and occlusive vascular disorders such as acute coronary syndrome or stroke (1). Despite lack of nucleus and genomic DNA, there are diverse types of RNAs in platelet, such as protein coding messenger RNAs (mRNAs), microRNAs (miRNAs), YRNAs and circular RNAs, which are inherited from their parent megakaryocytes (2–5). Platelets contain the essential machinery for processing miRNAs, which regulate the expression of proteins through complementary sequence recognition, binding and post-translational repression of mRNA transcripts (6, 7). The function of platelet miRNAs has gained widespread interest in the field of platelet biology since the platelet miRNA expression profile is closely associated with platelet biogenesis and activation. Recent studies have shown that platelets transfer and incorporate specific miRNAs into the recipient cells (8–11), raising the exciting possibility of potential therapeutic targets and disease biomarkers. In this review, we summarize and discuss the mechanistic roles of platelet miRNAs in circulation.

## Platelet mRNAs and miRNAs

MicroRNAs are generated either as transcripts that rely on transcription as the promoter for the host protein-encoding gene or by using their own promoter transcript in intergenic region of the genome (12). Anucleate platelets retain megakaryocyte-derived mRNAs (13) and their unique adaptive signals have evolved in maintaining the diversity of genes and proteins (14, 15).

With the use of high-throughput sequencing, Hélène Plé et al.  showed that based on the size of RNA, most of small RNAs found in platelets were miRNAs (16). Platelets have miRNAs, Dicer and Argonaute protein complexes, so pre-RNAs can be processed into miRNAs and control reporter transcripts. Analysed by the deep sequencing techniques, there were about 9,500 transcripts in the platelet of healthy subject (5, 17). Subsequent analysis of microarray and RNA sequencing have focused on non-healthy individuals, which correlated RNA profiles to specific human diseases (18–23). The stability of platelet miRNAs is unique, especially for the most abundant transcripts, suggesting that the platelet miRNome may be more stable than the miRNomes of other circulating cells or blood pools, such as plasma (24). It is noteworthy that almost half of miRNAs in microparticles, the major proportion of miRNAs in plasma, are produced by platelet (25). Numerous miRNAs have been found to be expressed in platelets and associated with cardiovascular diseases (CVD). Duisters RF et al. (24) reported that miR-30c repressed CTGF expression which was associated with ventricular fibrosis and heart failure. Tang, Y. et al. (26) found that miRNA–150 protected the mouse heart from ischemic injury by regulating EGR2, MYB and P2R × 7. McManus DD et al. (27, 28) demonstrated that miR-328 regulated CACNA1C and CACNB1 expressions, leading to atrial fibrillation and electrical remodeling. Willeit P et al. (29) showed that miR-342 regulated AKT1, glucose metabolism, apoptosis and cell proliferation, resulting in inflammatory stimulation of macrophages. Over the past decade, emerging evidence suggested that platelet miRNAs were potential regulators of platelet protein translation and expression as well as biomarkers for hematologic disease and platelet reactivity (4). In addition, considerable attention has be paid to the horizontal transfer of platelet miRNAs to the recipient cells.

## Platelets and Platelet-Derived Microparticles

Platelets are fragments of cytoplasm, which are derived from the megakaryocytes (29) of the bone marrow and lung (30, 31), then entered into the circulation. Circulating blood platelets have a lifespan of 8–10 days, and play a central role in hemostasis (32, 33). At the site of injury, platelets contact with many adhesion molecules, including collagen, which interacts with platelet surface receptors, leading to the adhesion and activation of platelets. Once activated, platelets release their bioactive components, such as ADP, serotonin and thromboxane A2, leading to further platelet recruitment, aggregation and plug formation (7). Platelet hyperreactivity contributes to the pathogenesis of thrombotic and occlusive vascular disorders, such as atherosclerosis, thrombotic cardiovascular disorders, occlusive thrombus or inflammation and cancer (34–38).

Microparticles (MPs), also referred to as microvesicles or more rarely ectosomes, are submicron fragments shed from stimulated plasma membrane or apoptotic cells (39). Platelet-derived MPs (PMPs) generated from more than 45% of plasma-borne MPs (39, 40). Based on the size of exosomes (<0.1 µm) and apoptotic bodies (>1 µm), PMPs (0.1–1 µm) can be distinguished from them. PMPs are shed from cytoplasmic membrane of platelet express the surface antigens, such as CD41 and CD62p (P-selectin) which are different from exosomes released from intracellular compartments (41).  Boilard et al. showed that platelets generated microparticles (0.1 µm) by stimulating the collagen receptor glycoprotein VI in arthritis pathophysiology (36). Moreover, recent studies have demonstrated that measurement of PMPs can be used as the marker for platelet activation (42, 43).

## The Horizontal Transfer of Platelet-Derived mRNA/miRNA Into the Vascular Cells

Upon activation, platelets can regulate the function of vascular cells by releasing the bioactive molecules. Platelet mRNAs are only associated with low levels of protein translation; however, platelets have a unique membrane structure that allows small molecules to pass through, so their cytoplasmic RNA can be delivered to nucleated cells (44). Antonina Risitano et al. demonstrated that coculture of platelet-like particles (PLPs) derived from megakaryoblastic cell line Meg-01 and human umbilical vein endothelial cells (HUVECs) led to the horizontal transfer of platelet RNA into HUVECs, and those incorporated RNA were functional in the recipient cells (44). Interestingly, platelets were also found to transfer specific miRNA to endothelial cells and regulate their gene expression of the endothelial cells. Benoit Laffont et al. found that platelets stimulated with 0.1 U/ml thrombin preferentially released MPs containing miR-223, and those MPs were internalized by HUVECs (8). Also, PMPs contain Argonaute 2 (Ago2)·miR-223 complexes, suggesting that the incorporated miRNAs were functional in the recipient HUVECs. Indeed, they found that platelet-derived miR-223 regulated two endogenous endothelial genes FBXW7 and EFNA1 at mRNA and protein levels (8).

The horizontal transfer of platelet RNA has also been reported in THP-1 cells (44). Antonina Risitano et al. found that the RNA of PLPs derived from Meg-01 was transferred to THP-1 cells, and those incorporated RNAs were functional in the recipient cells (44). Using microarray analysis of THP-1 cells showed that the genes globins (HGB1/HGB2) and globins (HBA1/HBA2) involved in transfer were up-regulated, but not altered in the control cells (44). In addition, after infused with wildtype platelets, the presence of TLR2-positive monocytes was found in TLR2-deficient mice treated with LPS, indicating that specific platelet RNAs are transferred and incorporated into leukocytes in vivo (44).

Benoit Laffont et al. demonstrated that PMPs containing miR-126–3 p was internalized by primary human macrophages (10). The incorporated miR-126–3 p suppressed the expression of four predicted mRNA targets of miR-126–3 p, and two of them were determined at the protein level. The effect of miR-126–3 p was further confirmed by expressing a neutralising miR-126–3 p sponge (10). Furthermore, PMPs induced the upregulation of 34 miRNAs with concomitant downregulation of 367 RNAs, including mRNAs encoding cytokines/chemokines chemokine (C-C motif) ligand 4 (CCL4), colony stimulating factor 1 (CSF1) and tumor necrosis factor (TNF) (10). These changes induced by PMPs were accompanied by a marked increase in the phagocytic capacity of macrophages (10).

Vascular smooth muscle cells (VSMCs) are the major component of the vascular wall and endure both ongoing damage and repair during atherosclerosis (45). During repair, newly migrated VSMCs tend to differentiate and frequently undergo apoptosis (46). A recent study has found that platelets may transfer miRNAs to VSMCs via platelet-derived exosomes (47). They also showed that in the murine model of carotid tandem stenosis, the levels of miR-21, miR-223 and miR-339 were associated with platelet activation (47). These miRNAs were elevated in the pooled mouse plasma exosomes before thrombosis, and were also found to be enriched in thrombin-stimulated platelet-derived exosomes in vitro (47). After cocultured VSMCs with platelet-derived exosomes, the expression of platelet-derived growth factor receptor-beta (PDGFRβ) in VSMCs was decreased (47). Platelet-derived exosomes also inhibited PDGF-stimulated VSMC proliferation. Furthermore, a decrease in PDGFRβ expression was observed in VSMCs around thrombotic areas in vivo (46). Despite lack of direct evidence showing the horizontal transfer of platelet miRNAs into VSMCs *in vivo*, these findings provide some clues for the possible role of platelet-derived exosomes containing miR-21, miR-223 and miR-339 in regulating the phenotypic switching of VSMCs in diseased condition (46).

## The Horizontal Transfer of Platelet-Derived mRNA Into the Hepatocytes

Marc Kirschbaum et al. showed that either platelets or PLPs derived from the MEG-01 stimulated the proliferation of HepG2 cells (9). They also observed platelet internalization into hepatocytes following a partial hepatectomy in mice (9). PLPs-derived RNA was detected in the cytoplasm of the HepG2 cells. The removal of PLPs-derived RNA by RNA-degrading enzymes partly blocked the stimulating effect of platelets on hepatocyte proliferation (9). Thus, platelets stimulate hepatocyte proliferation via the horizontal transfer of platelet RNA (9). Although no further evidence has been reported that miRNAs were involved in this process, it is plausible that activated platelet could transfer specific miRNAs to hepatocytes and participate into the regulation of liver regeneration.

## The Horizontal Transfer of Platelet-Derived mRNA/miRNA Into the Tumor Cells

Tumor growth is a major pathophysiological condition that may be affected by platelet MPs and associated miRNAs (7, 48). PMPs can directly stimulate the tumor growth through the release of potent growth factors in the tumor micro-environment (49). In 2015, Liang et al. first reported that platelet-secreted miRNA exacerbated lung cancer cell invasion (49). They found that miR-223 significantly increased in MVs derived from platelets of Non-small cell lung cancer patients (49). After cocultured with A549 cells, MVs derived from platelets delivered miR-223 into the recipient cells (49). The incorporated miR-223 exacerbated A549 cells invasion via targeting EPB41L3 (49).

Recently, James V. Michael et al. reported that platelet MPs suppressed tumor growth and induced tumor cell apoptosis (11). Solid tumor vasculature is highly permeable, allowing the possibility of PMPs-tumor interaction. They showed that PMPs infiltrated solid tumors in human and mice, thereby transferring platelet-derived miRNAs into tumor cells *in vitro* and *in vivo*, resulting in tumor cell apoptosis (11). PMPs transfusion suppressed the growth of both lung and colon carcinoma ectopic tumors, whereas blockade of miR-24 in tumor cells accelerated tumor growth. Par4 KO mice had the phenotype of reduced PMPs generation, and the tumor growth in Par4 KO mice was accelerated compared with wild type mice, indicating that PMPs play a pivotal role in the inhibition of tumor growth (11). The direct targets of PMPs-derived miR-24 in tumor cells were found to be the mitochondrial mt-Nd2 and a small noncoding nucleolar RNA Snora75 (11). The inhibition of mt-Nd2 and Snora75 resulted in mitochondrial dysfunction and growth inhibition of tumor cells (11). Thus, further studies are needed to determine the mechanistic role of the incorporated PMPs-derived miRNAs in the tumor progression.

## The Clinical Significance, Challenges and Limitations

Accumulating evidence shed light on the use of platelet-derived miRNAs as diagnostic markers for various diseases. Platelet-derived miRNAs have been associated with atrial fibrillation, coronary disease, heart failure and vascular disease (50). For example, miR-30c was associated with heart failure (24), miR-126 was associated with myocardial angiogenesis (28), miR-197 was associated with metabolic syndrome (51), miR-328 was associated with atrial fibrillation (27). Since the blood samples from patients are easily accessible, the identification of platelet-derived miRNAs in diseases will open up a new avenue for the clinical diagnosis and treatment.

As discussed above, many studies have reported that platelets, MPs and associated miRNAs can be transferred and incorporated into the various cells, and regulate the function of the recipient cells. However, there are still a number of concerns to be addressed. First, it is important to understand how platelets secretes specific miRNAs in response to different stimuli and pathological conditions, in order to precisely deliver therapeutic RNAs to target sites. Second, it is important to know how platelets and PMPs were selectively internalised by the recipient cells, in order to accurately deliver the platelets or PMPs to the specific cells, tissues, or injured sites. If all these unanswered questions could be addressed, using platelets and MPs as drug deliver system would be an efficient and site-specific approach for the treatment of platelet-associated diseases.

## Conclusion

**Figure 1:**
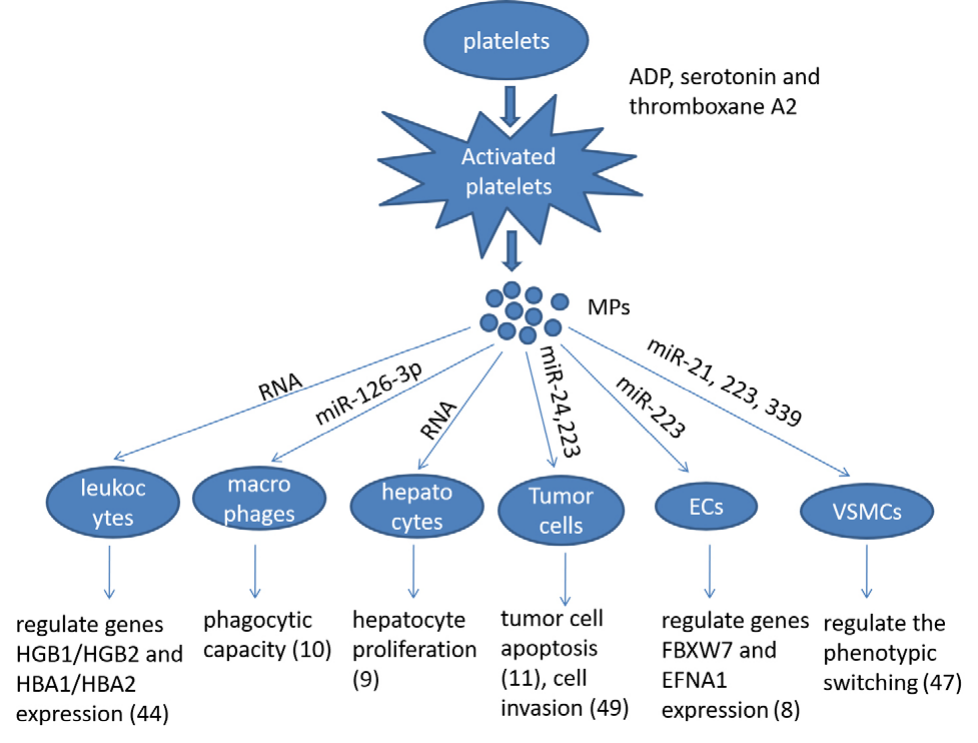
Summarizing the role of platelet microparticles (MPs) in cellular activation by the transfer of miRNAs. Ecs: endotheilal cells, VSMCs: vascular smooth muscle cells.

Platelets participate into multiple biological processes, such as inflammation, wound healing, maintenance of blood-lymph barrier and blood clotting where they provide a first and crucial line of defense against injury, thus maintaining normal homeostasis. As discussed above, recent advance in platelet biology demonstrates that activated platelets release the MPs containing abundant mRNA and miRNA, which are internalised by the recipient cells. The incorporated mRNA or miRNA can modulate the gene expression and regulate the function of the recipient cells, e.g., macrophages, ECs, leukocytes, VSMCs, hepotacytes, and tumor cells (Figure 1). Since the horizontal transfer of platelet miRNA may represent a novel form of cell-cell communication, which may participate in many pathophysiological processes, growing concern has been received in this field. However, many unanswered questions remain to be addressed about the specificity and selectivity of the horizontal transfer of miRNAs. Therefore, understanding the mechanism by which platelet interacts with other cells, and identifying which miRNAs are involved in the horizontal transfer would provide novel therapeutic targets and diagnostic markers for platelet-associated diseases.

## Author Contributions

LX wrote this manuscript. ZZ and WT were responsible for editing the manuscript. All authors accept responsibility for the entire content of this submitted manuscript and have approved its submission.

## Conflict of Interest Statement

The authors declare that the research was conducted in the absence of any commercial or financial relationships that could be construed as a potential conflict of interest.
